# Superb microvascular imaging for detecting neovascularization of carotid plaque compared with contrast-enhanced ultrasound: A protocol for systematic review and meta analysis

**DOI:** 10.1097/MD.0000000000021907

**Published:** 2020-08-28

**Authors:** Yang Zhou, Cong Wang

**Affiliations:** Ultrasound department of the First Affiliated Hospital of Dalian Medical University, China.

**Keywords:** carotid plaque, contrast-enhanced ultrasonography, intraplaque neovascularization, meta-analysis, superb microvascular imaging

## Abstract

**Background:**

Superb microvascular imaging (SMI) is a novel Doppler technique that depicts low velocity blood flow without the use of a contrast agent. Studies suggested that SMI may or may not detect neovascularization of carotid plaque with accuracy comparable to contrast-enhanced ultrasound. To gain clarity, a meta-analysis to systematically review and synthesize relevant data on the SMI for the detection of intraplaque neovascularization was undertaken.

**Methods:**

We will search PubMed, Web of Science, Cochrane Library, and Chinese biomedical databases from their inceptions to the June 31, 2020, without language restrictions. Two authors will independently carry out searching literature records, scanning titles and abstracts, full texts, collecting data, and assessing risk of bias. Review Manager 5.2 and Stata14.0 software will be used for data analysis.

**Results:**

This systematic review will investigate whether SMI is feasible on the detection of intraplaque neovascularization compared with contrast-enhanced ultrasound.

**Conclusion:**

Its findings will provide helpful evidence for the feasibility of SMI on the detection of intraplaque neovascularization.

**Systematic review registration:**

INPLASY202070097.

## 1 Introduction

Stroke is a common refractory disease that seriously endangers human health. Carotid artery atherosclerosis is a major risk factor for ischemic stroke. Intraplaque neovascularization (IPN) can promote the rapid progress of plaque, induce bleeding, and lead to plaque rupture. Plaque thrombi cause ischemic stroke while IPN is a major risk for plaque-related vascular events.^[1]^ Contrast-enhanced ultrasonography (CEUS) effectively visualizes IPN.^[2]^ Superb microvascular imaging (SMI) is as a novel Doppler technique that depicts low velocity blood flow without the use of a contrast agent.^[3]^ Studies suggested that SMI may or may not detect neovascularization of carotid plaque with accuracy comparable to CEUS.^[4–12]^ To gain clarity, a meta-analysis to assess the feasibility of SMI for the detection of IPN was undertaken compared with contrast-enhanced ultrasound.

## 2 Materials and methods

This study was conducted in accordance with the PRISMA (Preferred Reporting Items for Systematic Reviews and MetaAnalyses) guidelines and the protocol was registered in the INPLASY (INPLASY202070097).

### 2.1 Eligibility criteria

#### 2.1.1 Type of study

This study will only include high quality clinical cohort or case control studies that compare SMI with CEUS for evaluating IPN.

#### 2.1.2 Type of patients

The patients should be those who had undergone carotid atherosclerotic plaque formation.

#### 2.1.3 Intervention and comparison

IPNs of all patients were assessed with SMI and CEUS.

#### 2.1.4 Type of outcomes

The primary outcomes include a semi-quantitative scoring system, through which IPN was graded by means of both SMI and CEUS.

### 2.2 Search methods

PubMed, Web of Science, Cochrane Library, and Chinese biomedical databases will be searched from their inceptions to the June 31, 2020, without language restrictions. The search strategy for PubMed is shown in Table 1. Other online databases will be used in the same strategy.

**Table 1:**
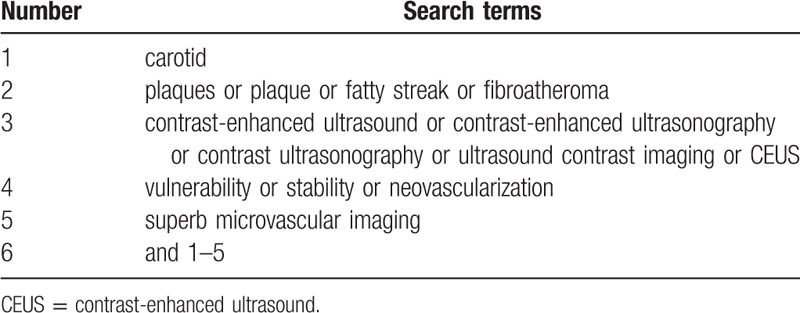
Search strategy sample of PubMed.

### 2.3 Data extraction and quality assessment

Two authors will independently select the trials according to the inclusion criteria, and import into Endnote X9. Then remove duplicated or ineligible studies. Screen the titles, abstracts, and full texts of all literature to identify eligible studies. All essential data will be extracted using previously created data collection sheet by 2 independent

authors. Discrepancies in data collection between 2 authors will be settled down through discussion with the help of another author. The following data will be extracted from each included research: year of article, first author's surname, sample size, number of intraplaque microvascular flow grades, number of every grade. The quality of selected studies will be independently evaluated according to a tool for the quality assessment of methodological index for non-randomized studies(MINORS). The MINORS criteria included 12 assessment items. Each of these items is scored as “yes” (2), “no” (0), or “unclear”(1). MINORS score ranged from 0 to 24; and score≥17 indicate a good quality. Any disagreements between 2 investigators will be solved through discussion or consultation by a 3rd investigator.

### 2.4 Statistical Analysis

The STATA version 15.1 software (Stata Corporation, College Station, TX) will be used for meta-analysis. We calculated the pooled summary odds ratio and its 95% confidence interval (CI). The Cochran *Q*-statistic and *I*^2^ test will be used to evaluate potential heterogeneity between studies.^[13]^ If the *Q*-test shows a *P* < .05 or *I*^2^ test exhibits > 50%, indicating significant heterogeneity, and the random effect model will be employed or if heterogeneity is not significant, the fixed-effects model was used. If it is possible, we will perform meta-analysis to analyze the pooled outcome data when acceptable homogeneity has been identified. Otherwise, we will conduct subgroup analysis to investigate potential causes for substantial heterogeneity among eligible studies. Sensitivity analysis will be performed to evaluate the influence of a single study on the overall estimate. We will use Begger funnel plots and Egger linear regression test to investigate publication bias.^[14]^

### 2.5 Ethics and dissemination

We will not obtain ethic documents because this study will be conducted based on the data of published literature. We expect to publish this study on a peer-reviewed journal.

## 3 Discussion

Atherosclerotic plaque formation occurs simultaneously with intraplaque angiogenesis.^[15]^ The development of summarized vascularity increases the probability of plaque-associated cardiovascular and cerebrovascular complications. Hemorrhage from intraplaque blood vessels can destabilize the plaque to promote rupture, further thrombosis, and cardiovascular events.^[16]^ Plaque neovascularization is a marker of plaque instability and predictor of cardiovascular and cerebrovascular diseases.^[17]^ Convenient, safe, and reproducible imaging methods to detect vulnerable arterial plaques provide crucial information supporting therapeutic interventions. Studies suggested that SMI may or may not detect neovascularization of carotid plaque with accuracy comparable to contrast-enhanced ultrasound. To gain clarity, in this study, we will perform a systematic review to summarize high-quality studies and to provide evidence on the evidence-based medical support for clinical practice.

## Author contributions

**Conceptualization:** Cong Wang.

**Data curation:** Cong Wang and Yang Zhou.

**Methodology:** Cong Wang.

**Writing – original draft:** Yang Zhou.

**Writing – review & editing:** Cong Wang.

## References

[1] Carlier S Kakadiaris IA Dib N Vasa vasorum imaging: a new window to the clinical detection of vulnerable atherosclerotic plaques. Curr Atheroscler Rep 2005;7:164 9. 15727733 10.1007/s11883-005-0040-2

[2] Staub D Patel MB Tibrewala A Vasa vasorum and plaque neovascularization on contrast-enhanced carotid ultrasound imaging correlates with cardiovascular disease and past cardiovascular events. Stroke 2010;41:41 7. 19910551 10.1161/STROKEAHA.109.560342

[3] Xie X Bai ZY Liu Y Value of superb micro-vascular imaging in diagnosing carotid artery vulnerable plaque. Zhongguo Yi Xue Ke Xue Yuan Xue Bao 2018;40:444 9. 30193595 10.3881/j.issn.1000-503X.10424

[4] Hoshino M Shimizu T Ogura H Intraplaque microvascular flow signal in superb microvascular imaging and magnetic resonance imaging carotid plaque imaging in patients with atheromatous carotid artery stenosis. J Stroke Cerebrovasc Dis 2018;27:3529 34. 30197167 10.1016/j.jstrokecerebrovasdis.2018.08.017

[5] Zamani M Skagen K Scott H Carotid plaque neovascularization detected with superb microvascular imaging ultrasound without using contrast media. Stroke 2019;50:3121 7. 31510899 10.1161/STROKEAHA.119.025496

[6] Ma L Ma XH Zhang YT Evaluation of neovascularization in carotid plaques of different risk levels: comparison of microvascular imaging and CEUS. Ningxia Med J 2018;40:307 10.

[7] Chen JJ Using SMI and CEUS to evaluate the neovascular in different thickness carotid atherosclerotic plaque. Master's thesis. Shijiazhuang: Hebei Medical University; 2016.

[8] Li TT Wang H The evaluation value of microvascular imaging and contrast-enhanced ultrasound in carotid atherosclerotic plaque. J Xinxiang Med Coll 2019;36:941 4.

[9] Cheng LG He W Zhang HX Superb microvascular imaging in evaluation of neovascularization in carotid plaque. Chin Med Imaging Technol 2015;31:647 50.

[10] Dong XY Li L Zhong YM Assessment of carotid artery plaque neovascularization by superb micro-vascular imaging and contrast-enhanced ultra-sound. J Medical Imaging 2018;28:16 9.

[11] Yan HR Clinical Research of Using Superb Micro-Vascular Imaging to Assess Neovascularization in Carotid Atherosclerotic Plaque Study on the Assessment of Neovascularization in Carotid Plaque by Ultramicro Flow Imaging. Master's thesis. Dalian: Dalian Medical University; 2018.

[12] He CY Fang B Li KL Studied and compared the diagnostic value of microvascular imaging and contrast-enhanced ultrasound in carotid plaque neovascularization. Jilin Med J 2018;39:2142 3.

[13] Zintzaras E Ioannidis JP HEGESMA: genome search meta-analysis and heterogeneity testing. Bioinformatics 2005;21:3672 3. 15955784 10.1093/bioinformatics/bti536

[14] Peters JL Sutton AJ Jones DR Comparison of two methods to detect publication bias in meta-analysis. JAMA 2006;295:676 80. 16467236 10.1001/jama.295.6.676

[15] Lee SH Lee JH Yoo SY Hypoxia inhibits cellular senescence to restore the therapeutic potential of old human endothelial progenitor cells via the hypoxia-inducible factor-1α–TWIST-p21 axis. Arterioscler Thromb Vasc Biol 2013;33:2407 14. 23928864 10.1161/ATVBAHA.113.301931

[16] Konstantino Y Wolk R Terra SG Non-traditional biomarkers of atherosclerosis in stable and unstable coronary artery disease, do they differ? Acute Card Care 2007;9:197 206. 17924231 10.1080/17482940701474486

[17] Chen RT Fu YC Wang W Intraplaque neovascularization and its influence on stability of atherosclerosis plaque. Chin J Arterioscl 2016;24:311 5.

